# Clinical and hospitalisation predictors of COVID-19 in the first month of the pandemic, Portugal

**DOI:** 10.1371/journal.pone.0260249

**Published:** 2021-11-19

**Authors:** Mariana Perez Duque, Neil J. Saad, Héloïse Lucaccioni, Cristina Costa, Geroid McMahon, Firmino Machado, Sooria Balasegaram, Rita Sá Machado

**Affiliations:** 1 Division of Epidemiology and Statistics, Directorate-General of Health, Lisbon, Portugal; 2 European Program for Intervention Epidemiology Training (EPIET), European Centre for Disease Prevention and Control (ECDC), Stockholm, Sweden; 3 Public Health Unit, ACeS Porto Ocidental, ARS Norte, Porto, Portugal; 4 Portugal Clinical Scholar Research Training, Harvard Medical School, Boston, MA, United States of America; 5 United Nations Relief and Works Agency for Palestine Refugees, Amman, Jordan; 6 Brigham and Women’s Hospital, Boston, MA, United States of America; 7 EPIUnit–Instituto de Saúde Pública, Universidade do Porto, Porto, Portugal; 8 Departamento de Ciências da Saúde Pública e Forenses e Educação Médica, Faculdade de Medicina da Universidade do Porto, Porto, Portugal; 9 Public Health England, London, United Kingdom; Azienda Ospedaliero Universitaria Careggi, ITALY

## Abstract

COVID-19 mainly presents as a respiratory disease with flu‐like symptoms, however, recent findings suggest that non-respiratory symptoms can occur early in the infection and cluster together in different groups in different regions. We collected surveillance data among COVID-19 suspected cases tested in mainland Portugal during the first wave of the pandemic, March-April 2020. A multivariable logistic-regression analysis was performed to ascertain the effects of age, sex, prior medical condition and symptoms on the likelihood of testing positive and hospitalisation. Of 25,926 COVID-19 suspected cases included in this study, 5,298 (20%) tested positive. Symptoms were grouped into ten clusters, of which two main ones: one with cough and fever and another with the remainder. There was a higher odds of a positive test with increasing age, myalgia and headache. The odds of being hospitalised increased with age, presence of fever, dyspnoea, or having a prior medical condition although these results varied by region. Presence of cough and other respiratory symptoms did not predict COVID-19 compared to non-COVID respiratory disease patients in any region. Dyspnoea was a strong determinant of hospitalisation, as well as fever and the presence of a prior medical condition, whereas these results varied by region.

## Introduction

The World Health Organization (WHO) declared coronavirus disease 2019 (COVID-19) a public health emergency of international concern on January 30, 2020 [1]. The first cases of severe acute respiratory syndrome coronavirus-2 (SARS-CoV-2) were reported on March 2, 2020 in Portugal [2]. SARS‐CoV‐2 infection mainly presents as a respiratory disease with flu‐like symptoms such as fever, cough, shortness of breath and fatigue, similar to symptoms reported when infected by other human coronaviruses [3,4]. However, recent findings on the clinical presentation of COVID-19 suggests that symptoms reported by patients other than respiratory or flu-like symptoms can occur in early phases of the infection [5,6]. Moreover, it was shown that COVID-19 symptoms cluster together in distinct groups [5].

Several factors can contribute to justify the heterogeneous clinical presentation of COVID-19 patients, between the different regions of Portugal, including socio-economic differences, disparities in disease burden, health outcomes and organisational differences in health provision [7,8]. In addition, socioeconomic status and social inequalities were already identified as drivers of disease, including most recently COVID-19 [8,9].

Therefore, we described the clinical characteristics of 25 926 suspected COVID-19 patients in the March 2020, first month of the pandemic, in mainland Portugal. We evaluated the clustering of symptoms of confirmed COVID-19 patients, to assess whether we observe a similar clustering as in previous studies. Finally, we determined the predictors of a positive SARS-CoV-2 test and hospitalisation, both nationwide and for each health region individually.

## Materials and methods

### Study design and setting

A prospective study was conducted among all COVID-19 suspected cases, tested in mainland Portugal, including public and private healthcare services. To assess clinical and hospitalisation predictors of COVID-19 in the first month of the pandemic in Portugal, we have restricted the study period to March 1^st^ to April 1^st^, 2020. COVID-19 cases were diagnosed based on the WHO interim guidance. A confirmed case of COVID-19 was defined as a positive result for SARS-CoV-2 virus on a real-time reverse-transcriptase–polymerase-chain-reaction (PCR) assay of nasal and/or pharyngeal swab specimens [10]. Until March 8^th^, the clinical criteria for testing was the presence of fever and/or cough, and/or shortness of breath, an epidemiological link with a confirmed COVID-19 case or recent travel history to an affected country was required. On March 9^th^, criteria were widened to include hospitalized cases with severe pneumonia with no other apparent cause. On March 26^th^, the criteria for testing were further expanded to include all cases of acute respiratory distress syndrome with cough and/or fever. The Directorate-General of Health (DGH) created an inner support service hotline to validate testing criteria for suspected cases by National Health Service (NHS) medical doctors. When a suspected case contacted the NHS, either in primary healthcare units or hospitals, a physician would first assess the presence of the relevant criteria and then would call the colleague on the hotline for further validation. Suspected cases could also call directly the NHS hotline, managed by NHS nurses, as the first contact with the healthcare services. in the latter case, the nurses would follow a similar further validation of the testing criteria. Given the comprehensiveness of the National Epidemiological Surveillance System (SINAVE), the compulsory notification of all COVID-19 suspected cases and our large sample size, we consider that our sample is representative of the population.

### Data sources and collection

We obtained all COVID-19 surveillance medical records and compiled data with laboratory test results, as reported to SINAVE between March 1^st^ and April 1^st^, 2020 [11,12]. All patients who filled the national COVID-19 case definition were tested for SARS-CoV-2 at no charge, regardless their residence legal status or having a private or occupational insurance. The surveillance report form was applied to all suspected cases tested, by the local public health officer. Only suspected cases with a laboratorial test result were included in the analysis. Suspected cases with no clinical data were excluded.

We extracted sociodemographic variables (age, sex, and health region), prior medical conditions and reported symptoms (fever, cough, shortness of breath, fatigue, myalgia, headache, arthralgia, sore throat, chest pain, diarrhoea, nausea, abdominal pain) at the time of the testing, registered in surveillance records from SINAVE. Clinical data on the case report form is coded as closed fields, with the option of *yes*, *no* and *unknown* answers. All surveillance records are electronic and national data-processing is under the coordination of the DGH. A team of clinicians and epidemiologists cleaned, reviewed and cross-checked the data.

### Outcomes

In our study, the primary outcome was test result for SARS-CoV-2 virus. Hospitalisation at the date of reporting was defined as a secondary outcome.

### Laboratory confirmation

Laboratory confirmation of SARS-CoV-2 was performed at the National Reference Laboratory before March 20, 2020, and subsequently in certified hospitals laboratories. PCR assays were performed in accordance with the protocol established by the WHO [10].

### Data analysis

Continuous variables were expressed as medians and interquartile ranges or simple ranges, as appropriate. Categorical variables were summarized as counts and percentages. To ascertain the differences between patients with a positive PCR test result, and those with a negative PCR test result, we used the Chi-squared test of independence to test differences between categorical variables. Differences between continuous variables were evaluated with the Student’s t-test and Wilcoxon test when appropriate.

We evaluated the clustering of symptoms among COVID-19 patients using a hierarchical clustering method (Ward linkage). The cluster analysis aims to classify objects and group them according to their similarities and in this method, objects are successfully integrated into a dissimilarity matrix computed by the data. The function *hclust* was used from R [13]. To assess uncertainty of the clustering method, a multiscale bootstrap sampling was used [14].

A multivariable backward stepwise logistic-regression analysis at the national level and for each of the five regions separately was performed to ascertain the effects of age, sex, prior medical condition and symptoms at the time of reporting (fever, cough, shortness of breath, sore throat, chest pain, headache, fatigue, myalgia, diarrhoea and abdominal pain) on the likelihood of testing positive and hospitalisation. Odds ratios and corresponding 95% confidence intervals were calculated.

A P-value of less than 0.05 was considered as a threshold. A new category for missing values on key variables was created (Supplementary material). All statistical analyses were performed using RStudio software, version 3.6.2 (R Foundation for Statistical Computing) and STATA software version 16.0 (StataCorp, College Station, USA).

### Ethical clearance

This study uses routine COVID-19 surveillance data. Routine data analysis of surveillance data is mandated by the Portuguese Ministry of Health to safeguard the health of the Portuguese people, for which the mandate has been given to the DGS [15]. The analysis of routine COVID-19 data falls within this mandate and therefore additional ethical approval was waived. All data were fully anonymised before this assessment and throughout the analysis confidentiality was assured.

### Patient and public involvement

No patients or members of the public were involved in the study design and implementation of the study.

## Results

### Sociodemographic and clinical characteristics of study population

A total of 25,926 COVID-19 suspected cases were included in this study, of whom 5,298 (20%) were PCR positive (Table 1). In the overall study population, the median age was 45 (IQR: 32 to 61), with 16% of the suspected cases aged 70 or more, and more than half (14,919 [58%]) were female. Most of the suspected cases were from the North region (49%), followed by Lisbon and Tagus Valley area (29%). COVID-19 patients were more likely to be older (median age 51 vs 44 years), male (59% vs 53%), more likely to be hospitalised (19% vs 11%) and have prior medical conditions (36% vs. 33%) compared to negative test patients (P-values all <0.001).

**Table 1:** Sociodemographic and clinical characteristics of study’s population by SARS-CoV-2 test result (N = 25,926).

	Overall, N = 25,926	Test negative, N = 20,628^1^	Test positive, N = 5,298^1^	P-value^2^
**Sociodemographic characteristics**				
**Female**	14,919 (58%)	12,085 (59%)	2,834 (53%)	<0.001
**Age, years**	45 (32, 61)	44 (31, 59)	51 (37, 65)	<0.001
**Health region**				<0.001
** North**	12,499 (49%)	9,337 (46%)	3,162 (60%)	
** Centre**	3,394 (13%)	2,744 (13%)	650 (12%)	
** Lisbon and Tagus Valley**	7,336 (29%)	6,102 (30%)	1,234 (23%)	
** Alentejo**	651 (2.5%)	604 (3.0%)	47 (0.9%)	
** Algarve**	1,411 (5.5%)	1,289 (6.3%)	122 (2.3%)	
**Clinical characteristics**				
**Hospitalised**	3,240 (13%)	2,210 (11%)	1,030 (19%)	<0.001
**Prior medical condition**	8,667 (33%)	6,786 (33%)	1,881 (36%)	<0.001
Fever* **(Y)**	10,662 (41%)	7,541 (37%)	3,121 (59%)	<0.001
Cough* **(Y)**	19,341 (75%)	15,499 (75%)	3,842 (73%)	<0.001
Shortness of breath* **(Y)**	6,393 (25%)	5,207 (25%)	1,186 (22%)	<0.001
**Sore throat (Y)**	6,906 (27%)	5,852 (28%)	1,054 (20%)	<0.001
**Chest pain (Y)**	3,132 (12%)	2,593 (13%)	539 (10%)	<0.001
**Myalgia (Y)**	7,366 (28%)	5,257 (25%)	2,109 (40%)	<0.001
**Fatigue (Y)**	5,665 (22%)	4,168 (20%)	1,497 (28%)	<0.001
**Headache (Y)**	7,618 (29%)	5,823 (28%)	1,795 (34%)	<0.001
**Nausea (Y)**	1,728 (6.7%)	1,302 (6.3%)	426 (8.0%)	<0.001
**Diarrhoea (Y)**	2,388 (9.2%)	1,770 (8.6%)	618 (12%)	<0.001
**Abdominal pain (Y)**	1,197 (4.6%)	963 (4.7%)	234 (4.4%)	<0.001

^*1*^ Statistics presented: n (%); median (IQR).

^*2*^ Statistical tests performed: Chi-square test of independence; Wilcoxon rank-sum test. P-values represented do not account for multiple testing.

* Symptoms included in the COVID-19 suspected case definition.

(Y)–yes.

Regarding clinical characteristics of COVID-19 suspected cases, cough, was the most common symptom (75%), followed by fever (41%) and headache (29%). Of the symptoms included in the case definition, only fever was proportionally higher among COVID-19 patients than the respective counterpart. Reported cough and shortness of breath was lower among negative test patients compared to COVID-19 patients (P<0.001). COVID-19 patients presented mostly with cough (73%), fever (59%), myalgia (40%) and headache (34%) at the time of notification. As regards to gastrointestinal (GI) symptoms, diarrhoea was the most common GI symptom in this study, accounting for 12% of the total enrolled patients with COVID-19. Concerning the other two reported GI symptoms, nausea and abdominal pain, only the latter was reported in a higher proportion among negative test patients (P<0.001).

### COVID-19 patients’ symptoms clusters

**Fig 1:**
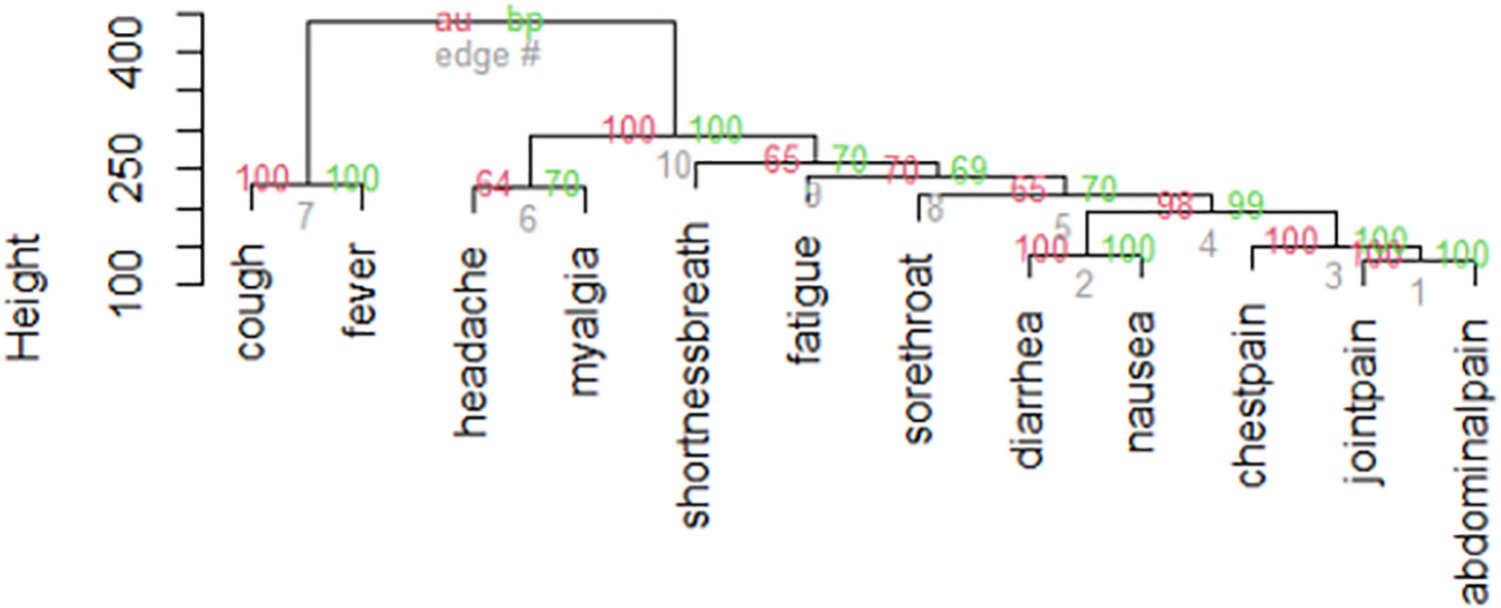
Symptoms cluster dendrogram of COVID-19 patients (n = 5,298). AU: Approximately unbiased p-value; BP: Bootstrap probability.

Fig 1 shows the hierarchical clustering of clinical signs and symptoms among COVID-19 patients. Symptoms were grouped into ten clusters, of which two main ones: one with cough and fever and another with the remainder. Joint pain and abdominal pain were the most nested symptoms, followed by GI symptoms of nausea and diarrhoea. Cough and fever, nausea and diarrhoea, abdominal and joint pain were grouped together in cluster duplets, which all had a high statistical clustering value, described as an approximately unbiased p-value (> 95%).

### Predictors for SARS-CoV-2 test positivity

**Fig 2:**
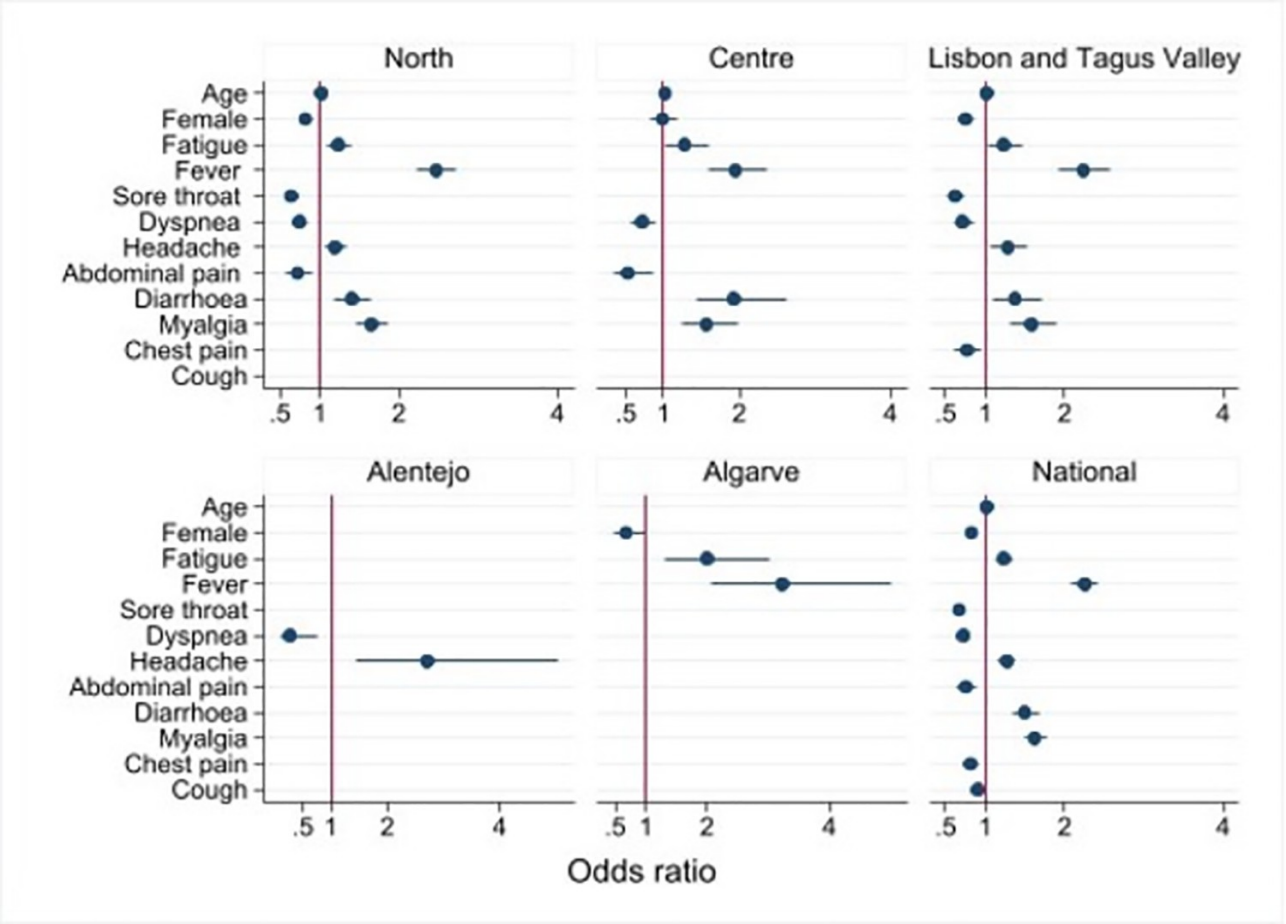
Predictors and 95% confidence intervals of SARS-CoV-2 virus test positivity using multivariate analysis, at the national level and by health region, among suspected cases of COVID-19, March-April 2020, (N = 25,926). Estimates for some predictors are missing because those were not retained by the model using a backwards stepwise model selection.

Predictors for SARS-CoV-2 test positivity and their corresponding odds ratios and 95% CI are shown in Fig 2. There was a higher odd of a positive test with increasing age (OR 1.02, 95% CI, 1.01 to 1.02; P<0.001), the presence of fever (OR 2.25, 95% CI, 2.09 to 2.42; P<0.001), fatigue (OR 1.24, 95% CI, 1.13 to 1.35; P<0.001), myalgia (OR 1.62, 95% CI, 1.48 to 1.77; P<0.001) and headache (OR 1.27, 95% CI, 1.16 to 1.39; P<0.001). The only GI symptom associated with a positive test result was diarrhoea (OR 1.49, 95% CI, 1.33 to 1.68; P<0.001). Females (OR 0.82, 95% CI, 0.77 to 0.88; P<0.001), respiratory symptoms, such as cough (OR 0.90; 95% CI, 0.83 to 0.98; P = 0.018), shortness of breath (OR 0.72; 95% CI, 0.66 to 0.79; P<0.001) and abdominal pain were associated with a negative test result for SARS-CoV-2, with no association found for the presence of joint pain. The analysis stratified by health region was consistent with the overall results (S2 Table). The predictor consistently associated with a positive test result for SARS-CoV-2 in all regions, except Alentejo, was fever.

### Predictors for hospitalisation among COVID-19 patients

**Fig 3:**
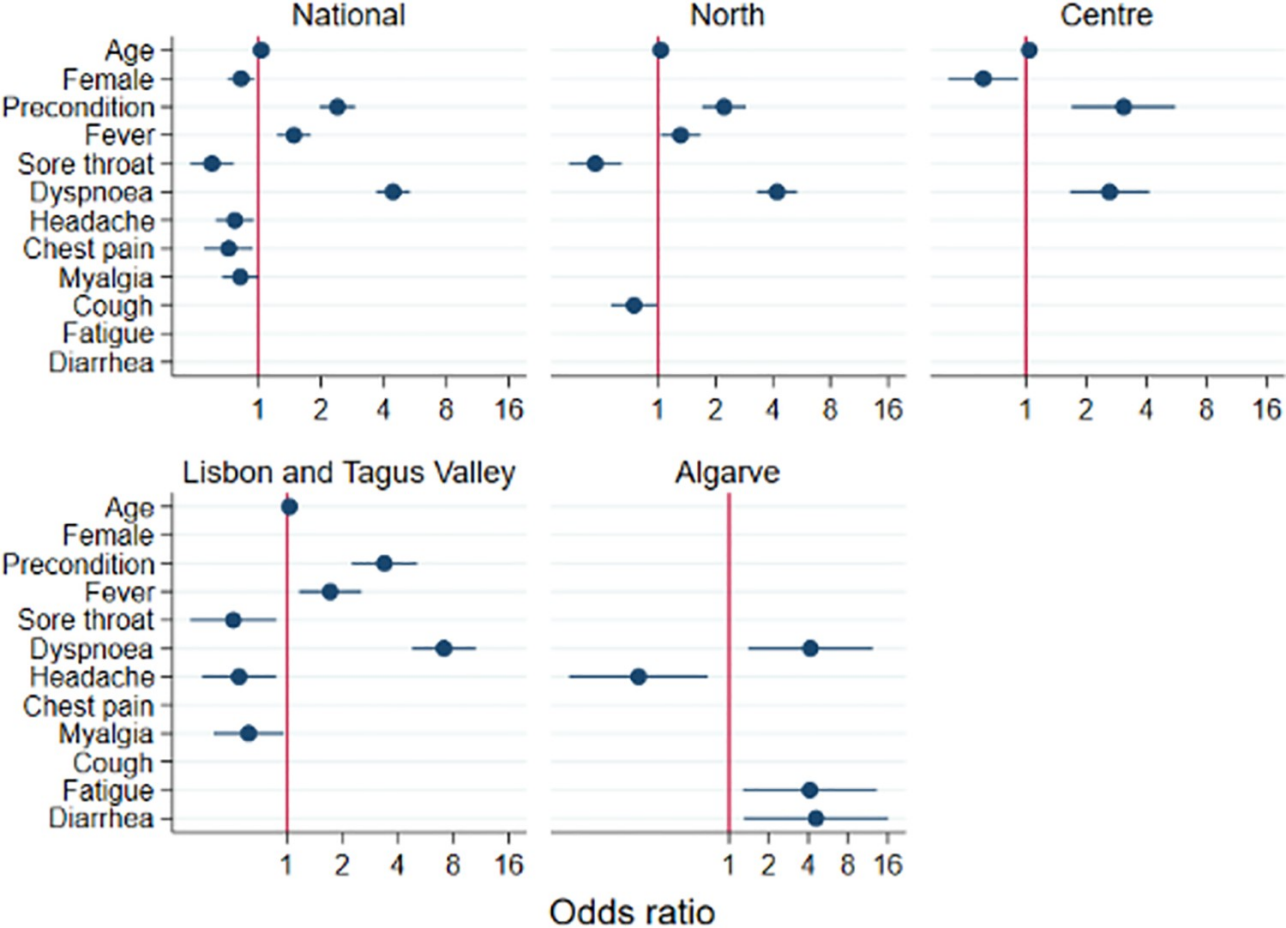
Predictors and 95% confidence intervals of hospitalisation using multivariate analysis, at the national level and by health region, among confirmed cases of COVID-19, March-April 2020 (N = 5,298). Estimates for some predictors are missing because those were not retained by the model using a backwards stepwise model selection.

Predictors for hospitalisation among COVID-19 patients and their corresponding odds ratios and 95% CI are shown in Fig 3. Among COVID-19 patients, the odds of being hospitalised increased with older age (OR 1.03, 95% CI 1.02 to 1.04; P<0.001), for those presenting with fever (OR 1.48, 95% CI 1.23 to 1.78; P<0.001), dyspnoea (OR 4.44, 95% CI 3.69 to 5.35; P<0.001), or those who reported a prior medical condition (OR 2.40, 95% CI 1.97 to 2.92; P<0.001). On the other hand, being female, having a sore throat, headache, myalgia or a chest pain was associated with a reduced odd of being hospitalised. These findings did vary by region, whereby some symptoms were no longer associated with the odds of hospitalisation. However, the presence of dyspnoea was consistently associated with a higher odds of hospitalisation in every region. In contrast, there was no symptom associated with a lower odds of hospitalisation in all regions. The presence of fatigue with an OR of 4.12 (95% CI 1.28 to 13.25; P<0.050) and diarrhoea, with an OR of 4.58 (95% CI 1.30 to 16.11; P<0.050) in Algarve region, was distinctive in relation to the other regions and national level.

## Discussion

In our large prospective study of 25 926 suspected cases of COVID-19, we presented the clinical symptoms associated with SARS-CoV-2 test positivity and hospitalisation during the first month of the pandemic in mainland Portugal. Our results also suggest that general systemic symptoms, such as fever, fatigue, myalgia, headache and also diarrhoea were associated with a positive test. Most of suspected cases who tested positive for SARS-CoV-2 virus had cough at the time of notification, however, cough did not predict COVID-19 compared to non-COVID respiratory disease. Instead, atypical symptoms, such as headache, fatigue, diarrhoea helped to differentiate patients who were more likely to be diagnosed with COVID-19 than those with other respiratory diseases. Dyspnoea was a strong determinant of hospitalisation, as well as fever and the presence of a prior medical condition, whereas these results varied by region.

Comparatively with other recent studies, we found that the clinical characteristics of SARS-CoV-2 infection in Portugal were similar to those from other previously reported studies [16,17]. Here, fever and cough were the dominant symptoms and, in comparison with the initial reporting from Wuhan, China, fever was present in a similar proportion of confirmed patients [17,18]. However, in Portugal, the most common GI symptom was diarrhoea, prevalent for 8% more than previously reported in other studies [19,20]. Headache, another key symptom associated with test positivity in our study, was found to be a strong predictor in line with reported literature [5,16,21]. Our findings affirm some of the risk factors for hospitalisation after infection with SARS-CoV-2. In particular, having dyspnoea increased the likelihood of hospitalisation, as shown in China [22].

An important aspect here is the identification of a difference in risk factors for test positivity and hospitalisation by region. These findings have not been previously reported for a nationwide study and could potentially be explained by differences in socio-economic conditions and health inequalities between regions [8,9]. Nevertheless, we acknowledge that further studies are needed, especially on genetic sequencing, as an added value to the epidemiological analyses made so far. It does, however, emphasise the importance of a robust surveillance system, with feedback to clinical and public health practitioners for the tailoring of the public health and clinical decision making and response.

Symptoms cluster analysis with an implementation of bootstrap analysis on a statistical model showed that some groups of symptoms cluster together and could help to predict a positive test among COVID-19 suspected cases [23].

A major strength of our study was the existence of a highly comprehensive surveillance system, which includes the whole population, providing a large study population, as all notified suspected COVID-19 patients were included in the study. Prospective data collection of both exposure and outcome data ensures temporality and therefore enhances the possibility of causal inferences. This study has some notable limitations. First, we might not have captured all signs and symptoms in this study, mainly lack of smell and taste, since during study’s period, these symptoms were not yet part of the report form. Moreover, we cannot rule out the role of genetic drivers that could explain different clinical manifestations as predictors of both test positivity and hospitalisation. Due to study’s design, we also cannot exclude the possibility of confounding, including the role of sociodemographic and economic factors on disease presentation, though all possible confounders and data available were included in the statistical analysis. The implementation of bootstrap sample approach tackles the uncertainty behind hierarchical cluster analysis, by randomly sampling elements of the data. The bootstrap replicates are obtained by repeatedly applying the cluster analysis to them. Although these methods are known for their application on phylogenetic analysis, they can be applicable to broad range of statistical problems, as we did in this study [14].

A better understanding of the spectrum of the disease during time is needed, and so additional study designs can give chronological perspective of clinical manifestations and their implication on predicting disease severity, as hospitalisation.

In this nationwide observational study involving COVID-19 suspected cases, we confirmed previous observations suggesting that presence of cough and other respiratory symptoms did not predict COVID-19 compared to non-COVID respiratory disease without regional variations. Dyspnoea was a strong determinant of hospitalisation, as well as fever and the presence of a prior medical condition, whereas these results varied by region. As community transmission of SARS-CoV-2 lasts, regions must be alert to miscellaneous clinical presentations of COVID-19 and subsequently test extensively for early case detection and treatment offer.

**Key points**

- Of 25,926 COVID-19 suspected cases included in this study, 5,298 (20%) tested positive
- Presence of cough and other respiratory symptoms did not predict COVID-19 compared to non-COVID respiratory disease patients in any region
- Dyspnoea was a strong determinant of hospitalisation, as well as fever and the presence of a prior medical condition, whereas these results varied by region
- As community transmission of SARS-CoV-2 lasts, regions must be alert to miscellaneous clinical presentations of COVID-19 and subsequently test extensively for early case detection and treatment offer

## Supporting information

S1 Fig Number of observations with missing data among study’s key variables (standardized). (TIF)

S1 Table Number of missing observations and proportion among study’s population (n = 25,926) on study variables. (DOCX)

S2 Table Multivariate analysis of predictors for SARS-CoV-2 test positivity, at the national level and by health regions. OR–Odds ratio, 95% CI– 95% confidence intervals.
(DOCX)
