# Supplementary figures and images for: Clinical and hospitalisation predictors of COVID-19 in the first month of the pandemic, Portugal
Source: PLoS One. 2021 Nov 19;16(11):e0260249. doi: 10.1371/journal.pone.0260249 (PMC8604361; doi:10.1371/journal.pone.0260249)

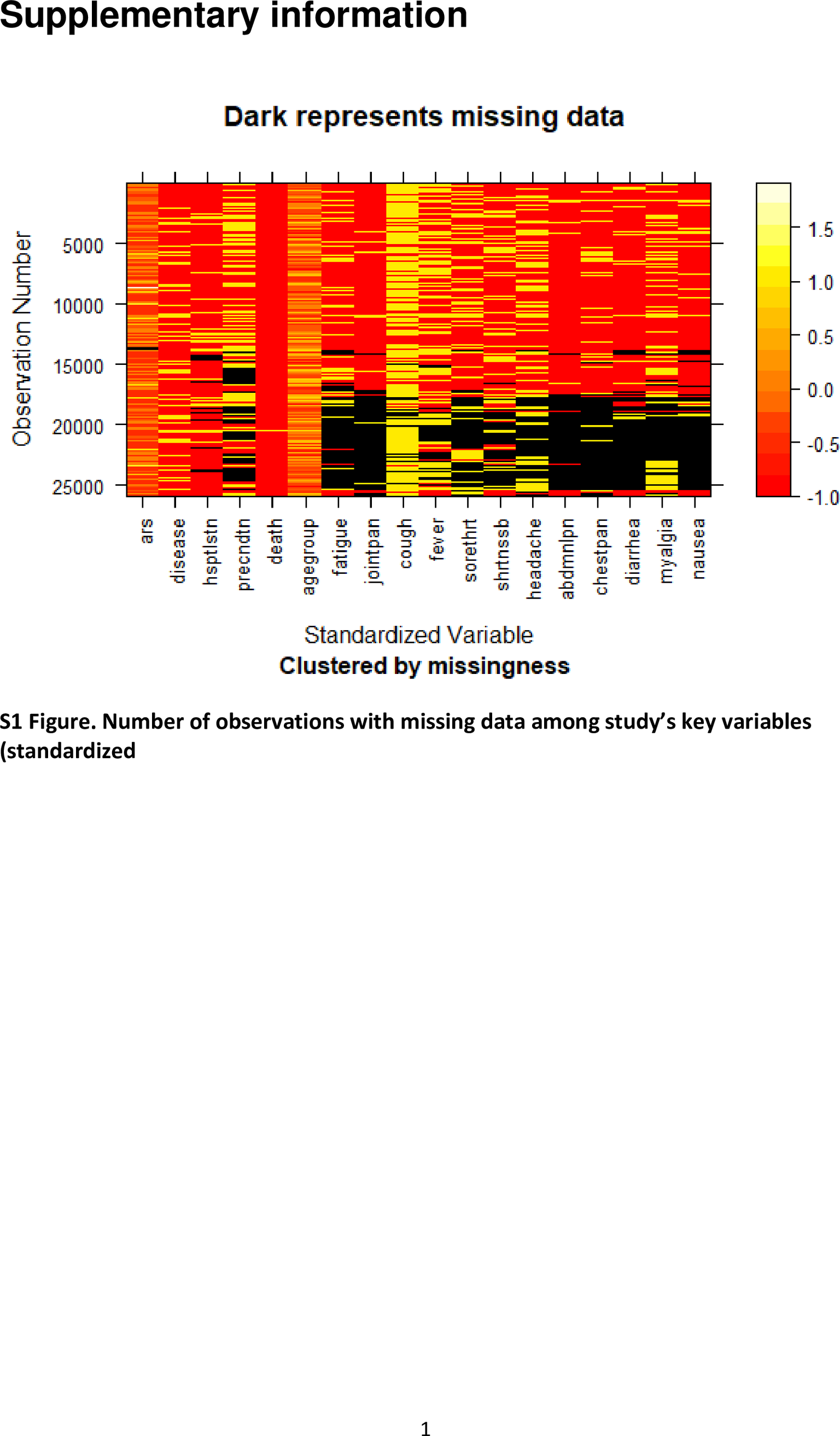

Supplement: S1 Fig — (TIF) [file pone.0260249.s001.tif]
