# Supplementary material for: Clinical and hospitalisation predictors of COVID-19 in the first month of the pandemic, Portugal
Source: PLoS One. 2021 Nov 19;16(11):e0260249. doi: 10.1371/journal.pone.0260249 (PMC8604361; doi:10.1371/journal.pone.0260249)
Supplement: S1 Table — (DOCX) [file pone.0260249.s002.docx]

**Supplementary information**

**S1 Table. Number of missing observations and proportion among study’s population (n= 25,926) on study variables**

| Variables | Test negative | Test positive |
| --- | --- | --- |
|  | **n (%)** | **n (%)** |
| Health region | 296 (1.4) | 1 (0.0) |
| Prior medical condition | 4 163 (20.2) | 1 482 (28.0) |
| Fatigue | 5 888 (28.5) | 1 741 (32.9) |
| Cough | 1 367 (6.6) | 480 (9.1) |
| Sore throat | 4 447 (21.6) | 1 656 (31.3) |
| Headache | 4 601 (22.3) | 1 403 (26.5) |
| Chest pain | 5 701 (27.6) | 1 840 (34.7) |
| Myalgia | 4 928 (23.9) | 1 291 (24.4) |
| Joint pain | 6 453 (31.3) | 2 028 (38.3) |
| Fever | 3 130 (15.2) | 647 (12.2) |
| Shortness breath | 3 921 (19.0) | 1 370 (25.9) |
| Abdominal pain | 5 943 (28.8) | 1 923 (36.3) |
| Diarrhoea | 5 493 (26.6) | 1 764 (33.3) |
| Nausea | 5 823 (28.2) | 1 867 (35.2) |
