# Supplementary material for: Clinical and hospitalisation predictors of COVID-19 in the first month of the pandemic, Portugal
Source: PLoS One. 2021 Nov 19;16(11):e0260249. doi: 10.1371/journal.pone.0260249 (PMC8604361; doi:10.1371/journal.pone.0260249)
Supplement: S2 Table — OR–Odds ratio, 95% CI– 95% confidence intervals. (DOCX) [file pone.0260249.s003.docx]

**Supplementary information**

**S2 Table. Multivariate analysis of predictors for SARS-CoV-2 test positivity, at the national level and by health regions**

| National | OR | 95% CI | p-value |
| --- | --- | --- | --- |
| Age | 1.02 | 1.01-1.02 | <0.001 |
| Female | 0.82 | 0.77-0.88 | <0.001 |
| Fever | 2.25 | 2.09-2.42 | <0.001 |
| Fatigue | 1.24 | 1.13-1.35 | <0.001 |
| Myalgia | 1.62 | 1.48-1.77 | <0.001 |
| Headache | 1.27 | 1.16-1.39 | <0.001 |
| Diarrhoea | 1.49 | 1.33-1.68 | <0.001 |
| Abdominal pain | 0.75 | 0.64-0.89 | 0.001 |
| Cough | 0.90 | 0.83-0.98 | 0.018 |
| Shortness of breath | 0.72 | 0.66-0.79 | <0.001 |
| Sore throat | 0.67 | 0.61-0.73 | <0.001 |
| Chest pain | 0.81 | 0.72-0.91 | <0.001 |
| North |  |  |  |
| Age | 1.02 | 1.02-1.02 | <0.001 |
| Female | 0.82 | 0.75-0.90 | 0.001 |
| Fever | 2.47 | 2.23-2.73 | <0.001 |
| Fatigue | 1.23 | 1.09-1.39 | 0.001 |
| Myalgia | 1.65 | 1.46-1.86 | <0.001 |
| Headache | 1.20 | 1.06-1.35 | 0.004 |
| Diarrhoea | 1.41 | 1.20-1.65 | <0.001 |
| Abdominal pain | 0.72 | 0.57-0.91 | 0.005 |
| Shortness of breath | 0.74 | 0.66-0.84 | <0.001 |
| Sore throat | 0.64 | 0.56-0.72 | <0.001 |
| Centre |  |  |  |
| Age | 1.02 | 1.01-1.02 | <0.001 |
| Fever | 1.95 | 1.59-2.38 | <0.001 |
| Fatigue | 1.28 | 1.01-1.61 | 0.038 |
| Myalgia | 1.57 | 1.25-1.97 | <0.001 |
| Diarrhoea | 1.93 | 1.42-2.62 | <0.001 |
| Abdominal pain | 0.53 | 0.33-0.85 | 0.008 |
| Shortness of breath | 0.71 | 0.57-0.89 | 0.003 |
| Lisbon and Tagus Valley |  |  |  |
| Age | 1.01 | 1.01-1.02 | <0.001 |
| Female | 0.75 | 0.66-0.85 | <0.001 |
| Fever | 2.24 | 1.94-2.58 | <0.001 |
| Fatigue | 1.23 | 1.03-1.47 | 0.020 |
| Myalgia | 1.58 | 1.32-1.89 | <0.001 |
| Diarrhoea | 1.37 | 1.10-1.71 | 0.005 |
| Chest pain | 0.77 | 0.61-0.96 | 0.020 |
| Shortness of breath | 0.71 | 0.60-0.85 | <0.001 |
| Sore throat | 0.62 | 0.52-0.74 | <0.001 |
| Alentejo |  |  |  |
| Headache | 2.71 | 1.46-5.02 | 0.002 |
| Shortness of breath | 0.27 | 0.09-0.78 | 0.015 |
| Algarve |  |  |  |
| Female | 0.68 | 0.46-0.99 | 0.045 |
| Fever | 3.22 | 2.08-4.99 | <0.001 |
| Fatigue | 2.00 | 1.33-3.06 | 0.001 |
